# Susceptibility to SARS‐Cov‐2 infection and risk for severe COVID‐19 in patients with prostate cancer on androgen deprivation therapy

**DOI:** 10.1002/ijc.34204

**Published:** 2022-07-26

**Authors:** Rolf Gedeborg, Stacy Loeb, Johan Styrke, Ritva Kiiski‐Berggren, Hans Garmo, Pär Stattin

**Affiliations:** ^1^ Department of Surgical Sciences Uppsala University Uppsala Sweden; ^2^ Department of Urology and Population Health New York University and Manhattan Veterans Affairs Medical Center New York New York USA; ^3^ Department of Surgical and Perioperative Sciences, Urology and Andrology Umeå University Umeå Sweden; ^4^ Department of Surgical and Perioperative Sciences, Anesthesiology and Intensive Care Medicine Umeå University Umeå Sweden; ^5^ Translational Oncology and Urology Research (TOUR), King's College London Guy's Hospital London UK

**Keywords:** androgen deprivation therapy, COVID‐19, GnRH agonists, prostate cancer, SARS‐CoV‐2

## Abstract

Androgen deprivation therapy (ADT) has been hypothesized to protect against COVID‐19, but previous observational studies of men with prostate cancer on ADT have been inconsistent regarding mortality risk from coronavirus disease 2019 (COVID‐19). Using data from the Prostate Cancer data Base Sweden (PCBaSe), we identified a cohort of 114 547 men with prevalent prostate cancer on the start of follow‐up in February 2020, and followed them until 16 December 2020 to evaluate the association between ADT and time to test positive for COVID‐19. Among men testing positive for COVID‐19, we used regression analyses to estimate the association between ADT and risk of COVID‐19‐related hospital admission/death from any cause within 30 days of the positive test. In total, 1695 men with prostate cancer tested positive for COVID‐19. In crude analyses, exposure to ADT was associated with a 3‐fold increased risk of both testing positive for COVID‐19 infection and subsequent hospital admission/death. Adjustment for age, comorbidity and prostate cancer risk category substantially attenuated the associations: HR 1.3 (95% CI: 1.1‐1.5) for testing positive for COVID‐19, and OR 1.4 (95% CI: 1.0‐1.9) for risk of subsequent hospital admission/death. In conclusion, although these results suggest increased risks of a positive COVID‐19 test, and COVID‐19‐related hospital admission/death in men on ADT, these findings are likely explained by confounding by old age, cancer‐associated morbidity and other comorbidities being more prevalent in men on ADT, rather than a direct effect of the therapy.

## What's new?

Increased susceptibility to COVID‐19 in men is suspected of being linked to androgen hormones. This idea is supported by evidence from men with prostate cancer, in which androgen deprivation therapy (ADT) was associated with a reduced risk of contracting COVID‐19. Here, in a Swedish cohort of men with prostate cancer, exposure to ADT was associated with an increased risk of COVID‐19 infection and subsequent hospital admission. These relationships, however, rather than being due to an effect of ADT on immune response to COVID‐19, are likely the result of confounding by older age and higher prevalence of cancer‐associated and other co‐morbidities.

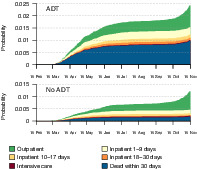

**Abbreviations**
ADT: androgen deprivation therapy
ATC code: anatomical Therapeutic Chemical code
CCI: Charlson comorbidity index
CI: confidence intervals
COPD: chronic obstructive pulmonary disease
COVID‐19: corona virus disease 2019
DCI: drug comorbidity index
GnRH: gonadotropin‐releasing hormone
ICD‐10: International Classification of Diseases, version 10
IQR: Interquartile range
MPA: Medical products agency (Swedish Government agency)
NPCR: National Prostate Cancer Register of Sweden
OR: Odds ratio
PCBaSe: Prostate Cancer data Base Sweden
PSA: Prostate‐specific antigen
RT‐PCR: Reverse‐transcriptase polymerase chain reaction
SARS‐CoV‐2: Severe acute respiratory syndrome coronavirus 2
SmiNet: Register on Surveillance of Notifiable Communicable Diseases

## 1 INTRODUCTION

Male sex is an independent risk factor for coronavirus disease 2019 (COVID‐19), which could be a consequence of sex‐specific differences in the response to viral infections.^1^ The mechanism for cellular entry of the coronavirus involves priming of the viral spike protein by the Type II Transmembrane Serine Protease (TMPRSS2).^2^ A role for the TMPRSS2 gene has also been proposed in the pathophysiology of prostate cancer. Androgens increase TMPRSS2 gene expression and this could therefore provide a potential explanation for the increased susceptibility to COVID‐19 in men.^3^ In line with this mechanism an observational study found that men on ADT had a lower risk of death from COVID.^4^ However, androgens influence host immune response through several different pathways, mostly with immunosuppressive effects.^5^

In a previous case‐control study of men with prostate cancer we found that men on ADT had an increased risk of death from COVID‐19 providing evidence against a protective effect of ADT against COVID.^6^ The increase in mortality from COVID‐19 was mainly related to old age, high comorbidity and more advanced prostate cancer in men on ADT. There was no clear evidence to support the hypothesis that ADT is associated with improved COVID‐19 outcomes.

In this present study, we aimed to clarify if the increased risk of death from COVID‐19 in men with prostate cancer on ADT is related to an increased risk of contracting the infection, or an increased risk of COVID‐19‐related hospitalization/death once infected.

## 2 METHODS

### 2.1 Study design and data sources

We performed a cohort study of men with prostate cancer in the National Prostate Cancer Register (NPCR) of Sweden, which includes comprehensive data on cancer features and treatment.^7^ Data for men in NPCR diagnosed with prostate cancer between 1 January 1998 and 31 December 2019 is linked to the Swedish Cancer Register,^8^ the Cause of Death Register,^9^ the Swedish Prescribed Drug Register (medications dispensed from all Swedish pharmacies),^10^ and the National Patient Register (hospital discharge and outpatient visit diagnoses),^11^ covering the period up to 16 December 2020. This has formed the Prostate Cancer data Base Sweden (PCBaSe) RAPID 2019.^12^

Data from PCBaSe can be linked to the Register on Surveillance of Notifiable Communicable Diseases (SmiNet) held by the Public Health Agency of Sweden,^13^ to which reporting notifiable diseases is mandatory for laboratory staff and treating clinicians. SmiNet includes each individual's personal identity number, date of disease occurrence, date of testing, date of positive tests, and diagnosis of the notifiable infectious disease. Linkage to SmiNet was performed to identify men diagnosed with COVID‐19 based on a positive reverse‐transcriptase polymerase chain reaction (PCR) during follow‐up.

PCBaSe was also linked to the Swedish Intensive Care Register (SIR), which contains information on the characteristics of patients admitted to an ICU, the reasons for admission, and severity scores indicating baseline risk,^14^ and comprehensive information on procedures, complications, treatment strategy and monitoring of organ dysfunction; in 2020, 81 out of all 83 Swedish ICUs reported data to the SIR.

### 2.2 Study population and follow‐up

For this present study, the main study cohort comprised a subset of men in PCBaSe RAPID 2019 who were alive at the start of follow‐up on 15 February 2020. Men were followed up to 16 December 2020 (end of the study period). Study outcomes were identified up until 17 November 2020 so that all men had at least 30 days of follow‐up following a positive PCR test.

### 2.3 Exposure to ADT

We identified exposure to ADT from prescriptions in the Swedish Prescribed Drug Register for the following medications any time prior to the start of follow‐up: the oral androgen receptor blockers bicalutamide (ATC codes L02BB03 and L02AE51) and flutamide (ATC code L02BB01; representing only 2% of men in this category), GnRH agonists (ATC code, L02AE), abiraterone (ATC code, L02BX03), and enzalutamide (ATC code, L02BB04). Adjuvant and neoadjuvant short‐term ADT were not included. Bicalutamide is according to the Swedish national treatment guidelines the ADT of choice for relapse after radical treatment. We previously reported high adherence to ADT is high and that it is rare for a man to discontinue his GnRH medication.^15^, ^16^, ^17^, ^18^, ^19^ Men who received 30 days of flare prophylaxis with bicalutamide in addition to GnRH agonist were classified as exposed to GnRH agonist only. We identified bilateral orchidectomy using procedure codes KFC10 or KFC15 in the National Patient Register. The exposure definition captured all men exposed to ADT, irrespective of indication or line of treatment. Men categorized as exposed to ADT were assumed to be exposed for the duration of follow‐up. We also categorized the duration of ADT exposure before start of follow‐up (0‐2 years, 2‐5 years, 5‐10 years and ≥10 years).

To reduce confounding by indication we excluded men with a prescription for the androgen receptor targeting drugs abiraterone and enzalutamide in the 90 days before the index date because of the strong association between use of these medications and an advanced stage of prostate cancer. In 18 of the 21 Swedish regions (covering 91% of the Swedish population on 31 December 2019), patients obtained GnRH agonists through community pharmacies; however, in three regions (Örebro, Värmland and Sörmland) GnRH agonists were given directly from the hospital, and were not captured in the Prescribed Drug Register. As a proxy for GnRH use in these three regions, we used a single prescription for bicalutamide under the assumption that it was given to prevent flare during the first month of GnRH treatment, as recommended by the Swedish national treatment guideline for prostate cancer.^20^

### 2.4 Outcomes

COVID‐19 was defined as having a positive test for COVID‐19 in SmiNet. We then categorized the severity of COVID‐19 based on healthcare use within 30 days after the positive test result as: death, admission to an ICU, admission to hospital and a duration of stay of 18 to 30 days, a hospital stay of 10 to 17 days, a hospital stay of 1 to 9 days, or out‐patient management throughout the 30 days follow‐up. These cut‐offs for length of stay were selected based on previous reports of length of hospital stay in older men with COVID‐19.^21^

### 2.5 Covariates

We used multiple variables to characterize prostate cancer risk category at diagnosis: cancer stage (TNM system),^22^ Gleason score, serum prostate‐specific antigen (PSA) and history of radical prostatectomy/radiotherapy. Prostate cancer severity was also determined from the interval between prostate cancer diagnosis and the start of follow‐up for the present study, and from fracture related to metastatic disease, or low‐energy fracture potentially related to general frailty, osteoporosis, or metastases. Opioid or a systemic corticosteroid prescriptions up to 6 months before the index date were used as an indicator of advanced cancer (Table S1). We calculated the Charlson Comorbidity Index from hospital discharge diagnoses in the National Patient Register during the 10‐year period before the start of follow‐up at the index date.^23^, ^24^ The National Patient Register was also used to obtain data on myocardial infarction, chronic obstructive pulmonary disease and diabetes in the 10‐year period before the index date (Table S2), and data on recent healthcare use. We measured the latter in two ways: first, as the sum of all unique main or secondary diagnosis codes from all outpatient visits in the 2 years before the index date, and second, as the sum of days of in‐hospital care for any reason in the 2 years before the index date. We also calculated a Drug Comorbidity Index (DCI) based on prescriptions in the Swedish Prescribed Drug Register within 365 days before the index date, categorizing medications by chemical subgroup, that is, the first five positions of the ATC code.^25^, ^26^

### 2.6 Statistical analysis

Characteristics of men at the time of prostate cancer diagnosis, as well as on the index date, were described using frequency counts and percentages for categorical variables, and medians with interquartile range for continuous variables. The cumulative incidence proportion of a hierarchical chain of potential events within 30 days of a positive test for COVID‐19 was calculated. We evaluated exposure to bicalutamide monotherapy separately from GnRH agonists and used men not exposed to any ADT as the reference group. In a complementary analysis we also estimated the association between duration of exposure to ADT and the risk for COVID‐19 infection. Cox proportional hazards regression was used to estimate crude and adjusted hazard ratios (HRs) for a positive test for COVID‐19, with 95% confidence intervals (CIs). We added covariates incrementally, representing potential confounding into the model, but with no selection of covariates based on the estimates. Person‐time was calculated from the index date 15 February 2020 to the date of a positive test result for COVID‐19 or end of PCR follow‐up on 17 November 2020. In the subgroup of men with a positive test for COVID‐19, we used logistic regression to estimate adjusted odds ratios (ORs) with 95% CIs for COVID‐19‐related hospital admission/ICU admission/death within 30 days of the test, adding grouped covariates incrementally into the model, but with no selection based on results. Age, DCI and the log PSA level at diagnosis were modeled as restricted cubic splines. Other numeric and count variables were stratified into categories; the Gleason score was grouped in five categories (2‐6, 7 [3 + 4], 7 [4 + 3], 8, 9‐10). Five models were generated, each with increasing adjustment for confounders to assess the impact on the crude OR. Covariates with missing values were imputed using multiple imputation with chained equations as implemented in the R package mice.^27^ Dichotomous variables were imputed with logistic regression, ordinal variables with ordinal regression and numerical variables with predictive mean matching.

## 3 RESULTS

The study cohort included 114 547 men with prevalent prostate cancer. In general, men on ADT, particularly those on a GnRH, were older at the time of prostate cancer diagnosis, had more locally advanced cancer, higher serum PSA levels and higher proportion of nodal and distant metastases compared with men not on ADT (Table 1). At the start of follow‐up, men on ADT had higher comorbidity scores, and a larger proportion of these men had signs of metastatic prostate cancer in terms of fracture, prescription of opioids and systemic corticosteroids and hospitalization with metastatic disease compared with men not on ADT. Primary radical treatment was less frequent among men on ADT.

**TABLE 1:** Characteristics of men with prevalent prostate cancer on 15 February 2020 in Prostate Cancer data Base Sweden (PCBaSe) RAPID 2019

	No ADT (N = 90 060)	Bicalutamide (N = 11 653)	GnRH (N = 12 834)
Characteristics at the time of prostate cancer diagnosis			
Age (years), median (IQR)	66.0 (61.0‐70.0)	71.0 (65.0‐77.0)	72.0 (66.0‐78.0)
Local clinical tumor stage at prostate cancer diagnosis, n (%)			
Localized (T1‐T2)	83 900 (93.2)	9545 (81.9)	7931 (61.8)
Locally advanced (T3‐T4)	4173 (4.6)	1871 (16.1)	3959 (30.8)
TX	1987 (2.2)	237 (2.0)	944 (7.4)
N0	25 963 (28.8)	3680 (31.6)	2943 (22.9)
N1	1017 (1.1)	381 (3.3)	1752 (13.7)
NX	63 080 (70.0)	7592 (65.2)	8139 (63.4)
M0^a^	89 659 (99.6)	11 406 (97.9)	10 013 (78.0)
M1	401 (0.4)	247 (2.1)	2821 (22.0)
Serum PSA at prostate cancer diagnosis (μg/L), median (IQR)	6.6 (4.6‐10.0)	11.0 (7.0‐20.0)	21.0 (9.8‐67.0)
Missing, n (%)	1831 (2.0)	110 (0.9)	116 (0.9)
Gleason score at prostate cancer diagnosis, n (%)			
2‐6	48 516 (53.9)	3160 (27.1)	2245 (17.5)
7 (3 + 4)	23 710 (26.3)	3116 (26.7)	2009 (15.7)
7 (4 + 3)	8378 (9.3)	2389 (20.5)	2246 (17.5)
8	4221 (4.7)	1336 (11.5)	2299 (17.9)
9‐10	3178 (3.5)	1164 (10.0)	3241 (25.3)
Missing^b^	2057 (2.3)	488 (4.2)	794 (6.2)
Primary treatment, n (%)			
Deferred treatment^c^	32 095 (35.6)	2758 (23.7)	1672 (13.0)
Radical prostatectomy	35 889 (39.9)	2606 (22.4)	1423 (11.1)
Radical radiotherapy	18 338 (20.4)	2697 (23.1)	1746 (13.6)
Bicalutamide	149 (0.2)	2947 (25.3)	1053 (8.2)
GnRH	242 (0.3)	194 (1.7)	6017 (46.9)
Orchidectomy	13 (0.0)	11 (0.1)	270 (2.1)
Missing^d^	3334 (3.7)	440 (3.8)	653 (5.1)
Any curative treatment, n (%)			
Yes	54 971 (61.0)	5419 (46.5)	3331 (26.0)
Characteristics on the start of follow up for the study (15 February 2020)			
Age (years), median (IQR)	73.3 (68.0‐77.8)	79.3 (74.0‐84.2)	79.9 (74.0‐85.3)
Time (years) since prostate cancer diagnosis, median (IQR)	6.3 (3.0‐11.0)	6.6 (3.6‐11.0)	6.9 (3.1‐11.8)
Duration of ADT (years), median (IQR)		3.3 (1.6‐6.0)	5.0 (2.3‐9.2)
Charlson comorbidity index, n (%)			
0	58 734 (65.2)	6260 (53.7)	6649 (51.8)
1‐2	24 013 (26.7)	3739 (32.1)	4238 (33.0)
3‐5	6632 (7.4)	1457 (12.5)	1698 (13.2)
≥6	681 (0.8)	197 (1.7)	249 (1.9)
Drug Comorbidity Index, median (IQR)	0.9 (0.2‐2.0)	1.5 (0.5‐2.9)	2.0 (0.9‐3.6)
History of myocardial infarction, n (%)	7500 (8.3)	1382 (11.9)	1549 (12.1)
History of diabetes mellitus, n (%)	8162 (9.1)	1494 (12.8)	1710 (13.3)
History of COPD, n (%)	4299 (4.8)	835 (7.2)	951 (7.4)
Number of diagnostic codes for specialist outpatient visits during the previous 2 years, median (IQR)	1.0 (0.0‐3.0)	2.0 (0.0‐3.0)	2.0 (1.0‐4.0)
Number of days in hospital during the previous 2 years, median (IQR)	9.0 (5.0‐15.0)	8.0 (5.0‐15.0)	8.0 (5.0‐13.8)
Fracture, N (%)	831 (0.9)	275 (2.4)	1491 (11.6)
Prescribed opioid, n (%)	8214 (9.1)	1220 (10.5)	2293 (17.9)
Prescribed systemic corticosteroid, n (%)	5591 (6.2)	868 (7.4)	2100 (16.4)
Diagnosis indicating metastatic disease, n (%)	1395 (1.5)	692 (5.9)	1965 (15.3)

Abbreviations: ADT, androgen deprivation therapy; COPD, chronic obstructive pulmonary disease; GnRH, Gonadotropin‐releasing hormone agonist; IQR, inter‐quartile range (25th to 75th percentile); PSA, prostate‐specific antigen.

^a^ Includes men for whom imaging was negative and men who had not undergone imaging (in accordance with TNM Classification of Malignant Tumors seventh Edition, edited by Leslie H. Sobin, Mary K. Gospodarowicz, Christian Wittekind, published in affiliation with the International Union Against Cancer [UICC]).

^b^ Also includes cases categorized as Gleason 7 without specification of 3 + 4 or 4 + 3.

^c^ Includes active surveillance, watchful waiting, and a previously used category for unspecified conservative treatment.

^d^ Also includes unspecified curative and noncurative treatment.

**FIGURE 1:**
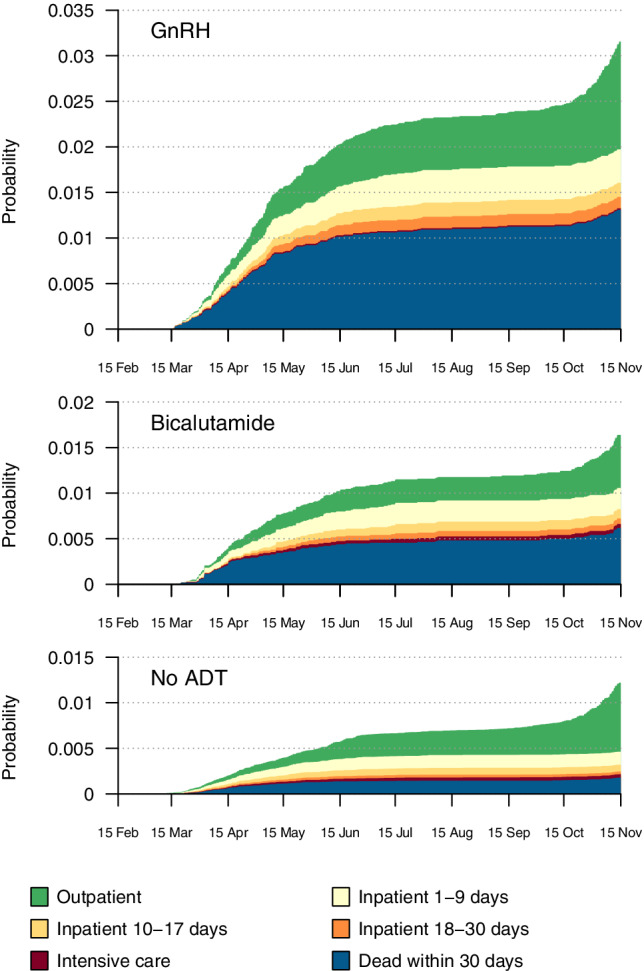
Stacked cumulative incidence proportions of positive COVID‐19 tests in 2020 categorized according to healthcare utilization within 30 days following the positive test, in a cohort of 114 547 men with prevalent prostate cancer.

In total, 1695 men tested positive for COVID‐19. Figure 1 shows the stacked cumulative incidence proportions of having a positive COVID‐19 test, and the proportions admitted to hospital or intensive care units, or dead from any cause within 30 days. There were two distinct time periods when the incidence increased, which corresponded to the two waves of the 2020 pandemic. Among men with a positive test for COVID‐19, 62% of those not on ADT and 37% of those on ADT survived at least 30 days and were not hospitalized. Mortality was higher in men exposed to ADT, but among men who survived at least 30 days after a positive test, 20% required hospitalization irrespective of ADT exposure. No further analysis of admissions to an ICU and need for respiratory support was possible because only seven men exposed to ADT were admitted to such a unit.

**FIGURE 2:**
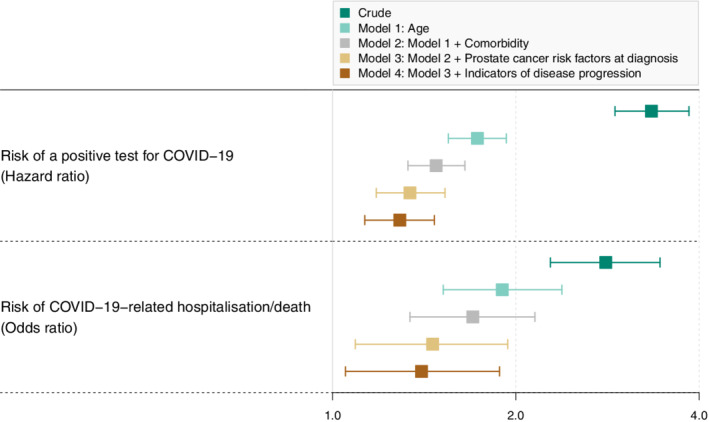
Forest plot of associations between exposure to any ADT and the risk of testing positive for COVID‐19 in 114 547 men with prostate cancer, and once infected, the risk those 1695 men required hospital admission. Estimated hazard ratios (upper panel) and odds ratios (lower panel) are represented by squares and 95% confidence intervals are represented by horizontal whiskers. The number of events, incidence rates, number of nonevents, and variables adjusted for in each step are described in Tables 2 and 3. ADT, androgen deprivation therapy.

In crude analyses, ADT exposure was associated with an increased risk for COVID‐19 infection (HR 3.3, 95% CI: 2.9‐3.8) (Figure 2 and Table 2). However, after adjustment for age, comorbidity, prostate cancer risk category and healthcare utilization, this association was substantially attenuated (adjusted HR 1.3, 95% CI: 1.1‐1.5). The crude association was much stronger with GnRH than bicalutamide, and this difference remained almost 2‐fold higher also after adjustment (Table 2).

**TABLE 2:** Hazard ratios with 95% confidence intervals for the association between ADT exposure and COVID‐19 infection in 114 547 men with prevalent prostate cancer followed from 15 February 2020.

Exposure	Events (N)	Incidence (Per 1000 person years)	Crude HR (95% CI)		Adjusted HR (95% CI) [Model 1: Age]		Adjusted HR (95% CI) [Model 2: Model 1 + Comorbidity^a^]		Adjusted HR (95% CI) [Model 3: Model 2 + Prostate cancer risk factors at time of diagnosis^b^]		Adjusted HR (95% CI) [Model 4: Model 3 + Indicators of disease progression^c^]	
No ADT	1099	16.4	1.00	(Ref.)	1.00	(Ref.)	1.00	(Ref.)	1.00	(Ref.)	1.00	(Ref.)
Any ADT	596	32.9	3.34	(2.91‐3.85)	1.73	(1.55‐1.93)	1.48	(1.33‐1.65)	1.34	(1.18‐1.53)	1.29	(1.13‐1.47)
Bicalutamide	191	22.0	2.29	(1.87‐2.80)	1.18	(1.00‐1.38)	1.07	(0.92‐1.26)	1.02	(0.86‐1.20)	1.00	(0.85‐1.18)
GnRH^d^	405	42.8	4.31	(3.68‐5.03)	2.25	(1.99‐2.54)	1.83	(1.62‐2.07)	1.79	(1.53‐2.09)	1.70	(1.45‐1.99)

Abbreviations: ADT, androgen deprivation therapy; CI, confidence interval; COPD, chronic obstructive pulmonary disease; GnRH, gonadotropin‐releasing hormone; HR, hazard ratio; PCBaSe, Prostate Cancer data Base Sweden.

^a^ Charlson Comorbidity Index + Drug Comorbidity Index + history of myocardial infarction, diabetes mellitus, COPD + number of days hospitalized + number of outpatient visits.

^b^ Local clinical tumor stage at diagnosis + Gleason score (2‐6, 7[3 + 4], 7[4 + 3], 8, 9‐10) + PSA + any curative treatment.

^c^ Years since prostate cancer diagnosis + hip/spine fracture or pathological fracture + opioid usage + systemic corticosteroid use + hospital discharge diagnoses indicating metastatic disease.

^d^ Includes men on GnRH analogues, GnRH antagonists, and men who underwent orchidectomy.

In a crude analysis, the association between ADT and increased risk of COVID‐19 infection was weaker in men with 0 to 2 years duration of ADT exposure (crude HR 1.7, 95% CI: 1.4‐2.0) compared with exposure ≥10 years (crude HR 2.6, 95% CI: 2.1‐3.1) (Table 4). This difference was substantially attenuated after adjustment for confounders, with only a small difference remaining between an exposure duration of 0 to 2 years (adjusted HR 1.23, 95% CI: 1.00‐1.51) and exposure for ≥10 years (crude HR 1.34, 95% CI:1.07‐1.69).

In a crude analysis of men who tested positive for COVID‐19, ADT exposure was associated with an almost 3‐fold increased risk of hospitalization or death, irrespective of ADT type (Table 3). In a multivariable analysis adjusted for age, comorbidity, prostate cancer risk category and healthcare utilization, the association was substantially attenuated, yet remained suggestive of an increased risk (adjusted OR 1.4, 95% CI: 1.0‐1.9).

**TABLE 3:** Odds ratios (ORs) with 95% confidence intervals (CIs) for the association between ADT exposure and hospitalization or death within 30 days after a positive test for COVID‐19 in men 114 547 men with prevalent prostate cancer

Exposure	Number of events	Nonevents (N)	Crude OR (95% CI)		Adjusted OR (95% CI) [Model 1: Age]		Adjusted OR (95% CI) [Model 2: Model 1 + Comorbidity^a^]		Adjusted OR (95% CI) [Model 3: Model 2 + Prostate cancer risk factors at time of diagnosis^b^]		Adjusted OR (95% CI) [Model 4: Model 3 + Indicators of disease progression^c^]	
No ADT	416	683	1.00	(Ref.)	1.00	(Ref.)	1.00	(Ref.)	1.00	(Ref.)	1.00	(Ref.)
Any ADT	376	220	2.81	(2.28‐3.45)	1.90	(1.52‐2.38)	1.70	(1.34‐2.15)	1.46	(1.09‐1.94)	1.40	(1.05‐1.88)
Bicalutamide	123	68	2.97	(2.15‐4.09)	2.13	(1.53‐2.98)	1.91	(1.36‐2.69)	1.67	(1.16‐2.40)	1.67	(1.15‐2.40)
GnRH^d^	253	152	2.73	(2.16‐3.46)	1.79	(1.39‐2.31)	1.60	(1.22‐2.09)	1.31	(0.94‐1.83)	1.22	(0.87‐1.72)

Abbreviations: ADT, androgen deprivation therapy; CI, confidence interval; COPD, chronic obstructive pulmonary disease; GnRH, gonadotropin‐releasing hormone; OR, odds ratio; PCBaSe, Prostate Cancer data Base Sweden.

^a^ Charlson Comorbidity Index + Drug Comorbidity Index + history of myocardial infarction, diabetes mellitus, COPD + number of days hospitalized + number of outpatient visits.

^b^ Local clinical tumor stage at diagnosis + Gleason score (2‐6, 7[3 + 4], 7[4 + 3], 8, 9‐10) + PSA + any curative treatment.

^c^ Years since prostate cancer diagnosis + hip/spine fracture or pathological fracture + opioid usage + systemic corticosteroid use + hospital discharge diagnoses indicating metastatic disease.

^d^ Includes men on GnRH analogues, GnRH antagonists, and men who underwent orchidectomy.

**TABLE 4:** Odds ratios (ORs) with 95% confidence intervals (CIs) for the association between duration of ADT exposure and COVID‐19 infection in 114 547 men with prevalent prostate cancer

Exposure	Events (N)	Incidence (Per 1000 person years)	Crude OR (95% CI)		Adjusted OR (95% CI) [Model 1: Age]		Adjusted OR (95% CI) [Model 2: Model 1 + Comorbidity^a^]		Adjusted OR (95% CI) [Model 3: Model 2 + Prostate cancer risk factors at time of diagnosis^b^]		Adjusted OR (95% CI) [Model 4: Model 3 + Indicators of disease progression^c^]	
No ADT	1099	16.4	1.00	(Ref.)	1.00	(Ref.)	1.00	(Ref.)	1.00	(Ref.)	1.00	(Ref.)
0‐2 years^d^	135	28.1	1.72	(1.44‐2.05)	1.56	(1.31‐1.87)	1.36	(1.13‐1.63)	1.24	(1.01‐1.52)	1.23	(1.00‐1.51)
2‐5 years^d^	173	29.8	1.82	(1.55‐2.14)	1.62	(1.37‐1.91)	1.41	(1.20‐1.66)	1.29	(1.08‐1.55)	1.25	(1.04‐1.50)
5‐10 years^d^	175	36.5	2.23	(1.90‐2.61)	1.88	(1.59‐2.22)	1.57	(1.33‐1.86)	1.43	(1.19‐1.71)	1.35	(1.13‐1.63)
10+ years^d^	111	41.7	2.55	(2.10‐3.10)	2.02	(1.64‐2.48)	1.66	(1.35‐2.04)	1.45	(1.16‐1.81)	1.34	(1.07‐1.69)

Abbreviations: ADT, androgen deprivation therapy; CI, confidence interval; COPD, chronic obstructive pulmonary disease; GnRH, gonadotropin‐releasing hormone; PCBaSe, Prostate Cancer data Base Sweden.

^a^ Charlson Comorbidity Index + Drug Comorbidity Index + history of myocardial infarction, diabetes mellitus, COPD + number of days hospitalized + number of outpatient visits.

^b^ Local clinical tumor stage at diagnosis + Gleason score (2‐6, 7[3 + 4], 7[4 + 3], 8, 9‐10) + PSA + any curative treatment.

^c^ Years since prostate cancer diagnosis + hip/spine fracture or pathological fracture + opioid usage + systemic corticosteroid use + hospital discharge diagnoses indicating metastatic disease.

^d^ Includes men on bicalutamide monotherapy, GnRH analogues, GnRH antagonists, and men who underwent orchidectomy.

## 4 DISCUSSION

In this large national population‐based cohort study, we followed over 100 000 men with prostate cancer during the first two waves of the COVID‐192020 pandemic in Sweden. In crude analyses, androgen deprivation therapy was associated with both the risk of contracting COVID‐19, and with an increased risk of COVID‐19‐related hospital admission/death. However, the crude relative measures of effect were substantially attenuated after multivariable adjustment for patient and disease‐related characteristics, suggesting that the observed associations are explained by confounding.

Previous epidemiologic studies on this topic have generally been based on small study populations, had limited ability to control  confounding, and generated mixed results.^28^, ^29^, ^30^, ^31^ Early during the COVID‐19 pandemic, some small noninterventional studies suggested that men with prostate cancer on ADT had lower rates of hospitalization, supplemental oxygen, intubation and death from COVID‐19.^4^, ^32^ Based on these early findings and the available evidence of a biologically plausible link between exposure to ADT and COVID‐19, several clinical trials were initiated to evaluate the benefit of ADT in men with COVID‐19.^33^ More recent noninterventional studies from other settings have not supported a protective effect of ADT on COVID‐19 outcomes.^34^, ^35^, ^36^

In a prior study among men with prostate cancer from Sweden, we compared relative excess mortality between those using ADT vs not using ADT between two time periods—during the first wave of the COVID‐19 pandemic (March‐June 2020) and the corresponding months in 2015 to 2019,^37^ and found no difference, including in analyses stratified by use of gonadotropin‐releasing hormone (GnRH) or bicalutamide monotherapy. In a subsequent nested case‐control study of COVID‐19‐related death in men with prostate cancer, we found no evidence to support the hypothesis that use of ADT is associated with a reduced risk of death from COVID‐19.^6^ Although a strong increased risk of COVID‐19 death was seen among men on ADT in unadjusted analyses, this was largely attenuated, albeit still elevated, after controlling for age (the strongest confounder), prostate cancer risk category, comorbidity, healthcare use, and other indicators of frailty and advanced cancer.

These three studies are consistent in suggesting against an association between use of ADT and risk of COVID‐19. No evidence of a protective effect was observed. There was essentially no excess mortality in men on ADT in adjusted analyses, while in the case‐control analysis there remained a small increased risk after adjustment for covariates. A similar residual increased risk was also observed in the present cohort analysis after adjustment for covariates. The study design evaluating excess mortality with a historical control period provides particularly good control of confounding by the stage of prostate cancer, since major changes in the population distribution of such patient characteristics are not expected over the recent years included in the study. This provides support for the assumption that the increase in risk that remained after adjustment in both the case‐control and the cohort analyses reflect residual confounding. We interpret our findings as ADT mainly being an indicator of patient frailty—including old age, high comorbidity and advanced prostate cancer stage—rather than a causal association between ADT and the COVID‐19 outcomes.

The apparent association between patient characteristics and risk of contracting COVID‐19 seen in our present study is particularly notable because it is likely that cancer patients considered themselves to be at high risk and are expected to have adhered particularly well to self‐isolation and other precautionary measures.^38^

A recent review of previous studies on the association between ADT and COVID‐19 outcomes in patients with prostate cancer identified some common and important study limitations.^39^ The authors raised concerns that studies failed to capture patients not regularly followed up outside the specialized hospital setting and could fail to include results from screening for COVID‐19. In that perspective, some notable strengths of our study are the large, population‐based, nation‐wide inclusion of men with prostate cancer, with exact person‐based linkage to other registries, thereby providing reliable ascertainment of all COVID‐19 test results, and near complete follow‐up of hospitalizations and deaths. Other concerns raised about previous studies were that the clinical data on use of ADT could be unreliable and that the duration of ADT exposure was unknown.^39^ In our study ADT exposure was reliably measured by use of in the Swedish Prescribed Drug Register. The majority of men in our study were using ADT at the time of exposure to COVID‐19, which should optimize the potential protective effect against COVID‐19. We analyzed the potential impact of the duration of ADT exposure prior to a COVID‐19 infection, and found it to be negligible after adjustment for confounding.

Other limitations of previous studies were their inability to reliably adjust for age and co‐ morbidities that critically contribute to infection outcomes.^39^ In the present study comorbidity was adjusted for both with the Charlson comorbidity index and a recently developed drug comorbidity index.^25^, ^26^, ^40^

Some remaining limitations of our study include that the information on prostate cancer stage at the time of the study did not include data on the Eastern Cooperative Oncology Group (ECOG) performance status, results from imaging, or treatment with chemotherapy. 35% of men not exposed to ADT were on deferred treatment and for those men there were no data on disease progression. The adjustment for cancer stage and the progression of the prostate cancer is therefore likely incomplete, causing potential residual confounding. Another potential limitation is that the availability of testing was more restricted initially during the first wave of COVID‐19.

In conclusion, androgen deprivation therapy was associated with an increased risk of contracting COVID‐19 in men with prostate cancer, and once infected, these men had an increased risk of hospital admission in crude analyses. These associations appeared to mainly reflect confounding by old age, high comorbidity and advanced prostate cancer, rather than a direct causal effect of ADT.

## AUTHOR CONTRIBUTIONS

The work reported in the paper has been performed by the authors, unless clearly specified in the text. Conceptualization: Pär Stattin, Hans Garmo and Rolf Gedeborg; Methodology: Hans Garmo; Writing—original draft preparation: Rolf Gedeborg; Writing—review and editing: Pär Stattin, Hans Garmo, Rolf Gedeborg, Stacy Loeb, Ritva Kiiski‐Berggren and Johan Styrke; Funding acquisition: Pär Stattin; Resources: Pär Stattin; Supervision: Pär Stattin.

## FUNDING INFORMATION

This study was supported by the Swedish Research Council (Grant number 2020‐05866). Stacy Loeb is supported by the Prostate Cancer Foundation and PCF Barry Family Center of Excellence at the Manhattan Veterans Affairs and the Edward Blank and Sharon Cosloy‐Blank Family Foundation. The funding source had no impact on the design, conduct, or interpretation of the study.

## CONFLICT OF INTEREST

The public health‐care administration for Region Uppsala in Sweden has, on behalf of the National Prostate Cancer Register, made agreements on subscriptions for quarterly reports from Patient‐overview Prostate Cancer with Astellas, Sanofi, Janssen and Bayer, as well as research projects with Astellas, Bayer and Janssen. Stacy Loeb reports previous equity in Gilead from a family member. Region Uppsala has, on behalf of NPCR, made agreements on subscriptions for quarterly reports quarterly reports from Patient‐overview Prostate Cancer with Astellas, Janssen and Bayer, as well as research projects with Astellas, Bayer and Janssen. All remaining authors have declared no conflicts of interest.

## ETHICS STATEMENT

The study was approved by the Swedish Ethical Review Authority: Dnr 2020‐03889. The requirement for informed consent was waived by the Ethical Review Authority.

## Supporting information


**TABLE S1** Operational definitions using the Anatomical Therapeutic Chemical (ATC) code
**TABLE S2**. Operational definitions using hospital discharge diagnoses coded with ICD‐10‐SE (Swedish clinical modification).

## Data Availability

Data used in the present study was extracted from the Prostate Cancer Database Sweden (PCBaSe), which is based on the National Prostate Cancer Register (NPCR) of Sweden and linkage to several national health‐data registers. Use of the data from national health‐data registers is further restricted by the Swedish Board of Health and Welfare and Statistics Sweden which are Government Agencies providing access to the linked health‐care registers. The data can, however, be retrieved after application made to any of the steering groups of NPCR and PCBaSe. The corresponding author can facilitate access to the data that support the findings of this study upon reasonable request. For detailed information, please see www.npcr.se/in-english, where registration forms, manuals and annual reports from NPCR are available alongside a full list of publications from PCBaSe. The code used for the present study analyses can be provided on request (contact hans.garmo@rccmellan.se).
