# Supplementary material for: Susceptibility to SARS‐Cov‐2 infection and risk for severe COVID‐19 in patients with prostate cancer on androgen deprivation therapy
Source: Int J Cancer. 2022 Jul 26:10.1002/ijc.34204. Online ahead of print. doi: 10.1002/ijc.34204 (PMC9349425; doi:10.1002/ijc.34204)
Supplement: Supplementary file 1 — TABLE S1 Operational definitions using the Anatomical Therapeutic Chemical (ATC) code TABLE S2. Operational definitions using hospital discharge diagnoses coded with ICD‐10‐SE (Swedish clinical modification). [file IJC-9999-0-s001.pdf]

Supplementary online material to:

Susceptibility to SARS-Cov-2 Infection and Risk for Severe COVID-19 in Patients with Prostate Cancer on Androgen Deprivation Therapy

Rolf Gedeborg, Stacy Loeb, Johan Styrke, Ritva Kiiski-Berggren, Hans Garmo, Pär Stattin

eTable 1. Operational definitions using the Anatomical Therapeutic Chemical (ATC) code

Condition	Operational definition
Opioid	N02A
Systemic corticosteroids	H02AB

eTable 2. Operational definitions using hospital discharge diagnoses coded with ICD-10-SE (Swedish clinical modification).

Condition	Operational definition
myocardial infarction	I21, I22, or I252
diabetes	E100-149
chronic obstructive pulmonary disease (COPD)	I278-279, J40-47, J60, J61-J67, J684, J701, or J703
Fracture potentially indicating frailty and/or more advanced prostate cancer stage	C795, G55, M485, M844, M495, M907, S220, S221, S320-326, S327, S328, or S720-722
Metastatic disease	C77, C78, or C79 as main diagnosis
